# Digital Follow-Up After Elective Laparoscopic Cholecystectomy: A Feasibility Study

**DOI:** 10.1007/s00268-022-06684-w

**Published:** 2022-08-11

**Authors:** Prita Daliya, Jody Carvell, Judith Rozentals, Dileep N. Lobo, Simon L. Parsons

**Affiliations:** 1 Trent Oesophago-Gastric Unit, Nottingham University Hospitals NHS Trust, City Hospital Campus, Hucknall Road, Nottingham, UK; 2 Gastrointestinal Surgery, Nottingham Digestive Diseases Centre and National Institute for Health Research (NIHR) Nottingham Biomedical Research Centre, Nottingham University Hospitals NHS Trust and University of Nottingham, Queen’s Medical Centre, Nottingham, UK; 3 MRC Versus Arthritis Centre for Musculoskeletal Ageing Research, School of Life Sciences, University of Nottingham, Queen’s Medical Centre, Nottingham, UK

## Abstract

**Background:**

Although recommendations exist for patients to be offered a post-operative helpline or telephone follow-up appointment at discharge after cholecystectomy, implementation of these is resource-intensive. Whilst the benefits of telephone follow-up are well documented, the use of digital modalities is less so. We aimed to identify if digital follow-up (DFU) was equivalent to routine care with telephone follow-up (TFU), for patients undergoing elective laparoscopic cholecystectomy.

**Methods:**

All patients listed for elective laparoscopic cholecystectomy between August 2016 and March 2018 were offered routine post-operative care (TFU or no follow-up) or DFU at a tertiary referral centre in Nottingham.

**Results:**

Of 597 patients undergoing laparoscopic cholecystectomy, 199 (33.3%) opted for TFU, and 98 (16.4%) for DFU. DFU was completed for 85 (86.7%) participants and TFU for 125 (62.8%), *p* < 0.0001. Over 5 times as many patients who chose TFU missed their appointment compared to DFU (5.6% vs. 30.9%, *p* < 0.001). At 30-days post-operatively, patients undergoing TFU had significantly more post-operative wound infections identified then those undergoing DFU (17.6% vs 5.9%, *p* = 0.01). However, this did not impact the incidence of 30-day readmissions between groups (7.2% TFU vs. 7.1% DFU). No complications were missed by either the DFU or TFU modalities. DFU was completed significantly earlier than TFU (median 6 days vs. 13.5 days, *p* = 0.001) with high patient acceptability, identifying complications and alerting clinicians to those patients requiring an early review.

**Conclusion:**

This feasibility study has demonstrated that digital follow-up is an acceptable alternative to telephone follow-up after elective laparoscopic cholecystectomy.

**Supplementary Information:**

The online version contains supplementary material available at 10.1007/s00268-022-06684-w.

## Introduction

Over 60,000 cholecystectomies are performed in the UK each year [1], with laparoscopic cholecystectomy being performed predominantly as a day-case procedure [2]. Whilst most institutions no longer offer routine post-operative follow-up, there are national guidelines recommending that either a post-operative helpline or telephone follow-up (TFU) appointment is provided at discharge [3]. The significant benefits of TFU are well documented, including increased patient satisfaction [4–8], reduced patient anxiety [4], reduced expenses and travel time for patients to reach hospital outpatient follow-up appointments [7], and reduced hospital costs in running a physical outpatient clinic service [5, 9]. Loss of follow-up also precludes necessary feedback for surgeons and a missed opportunity to guide learning and a change in practice [10].

Telemedicine and TFU, although less resource-intensive than physical follow-up appointments, still requires the use of trained health care professionals when compared with no follow-up, resources which are already stretched in the current National Health Service (NHS) and could be better used [9, 11].

Prior to Covid-19, the NHS aimed to deliver more health care remotely through the introduction of new technologies such as the NHS App [12]. This enables health care professionals in secondary care to communicate with patients and share information on their mobile devices at a time and place of their choosing. Integrating care locally [13] continues to be a priority and is even more important since the start of the pandemic. Despite advances in modern technology, little research has been undertaken to explore alternative modalities for post-operative follow-up, especially given that automated follow-up can minimise staffing, and the necessity for fixed appointment times.

The aim of this study was to identify if digital follow-up (DFU) using an online platform, was equivalent to routine care with telephone follow-up (TFU), for patients undergoing elective laparoscopic cholecystectomy. Primary objectives were to identify the incidence of 30-day post-operative complications identified by DFU compared with TFU. Secondary objectives were to identify the incidence of 30-day readmissions, reoperations, and the incidence of missed complications, in addition to follow-up compliance between groups.

## Methods

### Study design and setting

This prospective cohort study was conducted in a large NHS trust with tertiary centre capabilities. All patients listed for elective laparoscopic cholecystectomy between August 2016 and March 2018 were given free choice on whether they opted to use post-operative DFU or routine post-operative care. Routine care included TFU at the Nottingham City Hospital site, and no follow-up at the Queen’s Medical Centre and Circle Treatment Centre sites. The study was conducted in accordance with the Strengthening the Reporting of Observational Studies in Epidemiology (STROBE) guidelines [14].

### Study groups

Participants were recruited to participate in this study on referral to hospital for management of symptomatic gallstones. On receiving study information either postally or in person, each participant indicated their consent to participate digitally.

### Digital follow-up

Access to the DFU survey was automated once participants had confirmation of an operation date and were discharged from hospital. Internally coded information technology links with the Nottingham University Hospitals NHS Trust (NUH) Patient Administration System (PAS) and the online platform meant that admission and discharge dates for surgery were inputted automatically. The entire process was overseen by a researcher to ensure no system glitches throughout.

Study participants registering for DFU received a system-automated email to complete a digital survey at 7 days post-operatively, and one further reminder for non-responders at day 10.

The 7-day follow-up survey (Table 1) consisted of 12 questions in six categories. Each question was allocated a score, with a score of 3 or more in any one category, or an overall score of greater than 9 from the total of all 12 questions coded to trigger a call-back. Although participants were not informed of their score, they were immediately informed of the outcome of their score by one of two automated videos:

**Table 1:** Seven-day follow-up survey

Categories	Questions		Possible responses	Score
1	1	How would you rate your general well-being since your operation?	Better than before my operation	0
			Almost back to normal	0
			Back to normal	0
			Worse than before my operation, but getting better	1
			Worse than before my operation, and not getting better	3
2	2	Have you had to seek medical advice (from hospital, your GP, or a nurse) regarding anything to do with your operation since you were discharged from hospital care?	No	0
			Yes	1
	3	Is the problem better now?	No	2
			Yes	0
3	4	How much pain are you in following your operation?	None	0
			Mild	0
			Moderate	1
			Severe	2
	5	Which painkillers are you still currently needing for pain after your operation?	None	0
			Paracetamol	0
			Ibuprofen/co-codamol	0
			Tramadol/codeine/dihydrocodeine	1
			Morphine of similar	2
4	6	Bowel motions (stool) can change after an operation. Which term best describes them now?	Normal	0
			Loose (diarrhoea)	1
			Hard (constipation)	1
			Not been able to pass stool or flatus (wind from the back passage) since the operation	3
5	7	Have your stools become pale since your operation (yellow or white)?	No	0
			Yes	1
	8	Have you noticed any yellow discolouration of your eyes/skin (jaundice)?	No	0
			Yes	1
	9	What colour is your urine?	Normal	0
			Clear	0
			Dark	1
6	10	Are any of your wounds red and hot?	No	0
			Yes	1
	11	If you have discharge from your wound/s, is the discharge volume such that you are having to change your dressings 2 or more times daily?	No discharge	0
			No	0
			Yes	1
	12	What colour is the discharge from your wound/s?	No discharge	0
			Clear	0
			Red	0
			Yellow	1
			Green	3
			Brown	3

Video 1: Reassured and discharged:

Participants were provided with a downloadable patient recovery information sheet and contact details for a specialist nurse should they need.

Video 2: Telephone call-back within 24 h:

Participants were informed they would receive a telephone consultation within 24 h. They were also provided with a downloadable patient recovery information sheet and contact details for a specialist nurse.

Participant scores following completed DFU surveys were forwarded to the trial researcher who was then able to track responses and conduct TFU where appropriate.

### Telephone follow-up

Participants who opted for TFU were called between post-operative day 10 and 14 by one of three surgical care practitioners (SCPs). These were pre-booked appointment slots arranged with the patient on discharge from hospital. SCPs are trained health care professionals with prior registration as either an operating department practitioner or registered nurse with more than 5 years of post-registration experience.

Prospective audit was conducted for all patients. Data on complications, readmissions, reoperations, and mortality were taken from patient clinical notes, the PAS record of hospital attendances, and participant interview. Information on attendances to primary care, walk-in centres and other hospitals was, therefore, limited.

### Outcomes

The outcome measures monitored included 30-day post-operative complications, 30-day readmissions, re-operations, and missed post-operative complications, and follow-up compliance.

### Variables

Data on patient and hospital characteristics were collected, including patient age, sex, Charlson comorbidity index (CCI) [15], body mass index (BMI), and total length of hospital stay.

### Statistical analysis

Data were analysed using GraphPad Prism® version 8.3.0 (GraphPad Software LLC, San Diego, CA, USA.). Differences between groups were evaluated using either Fisher’s exact test or chi-squared tests for categorical variables and Mann–Whitney tests for continuous variables. Differences were considered significant at *p* < 0.05.

### Ethics

This study was a part of PhD project sponsored by NUH, through a collaboration with EIDO Healthcare Limited and The Royal College of Surgeons of England. The study proposal was appraised by the confidentiality advisory group: 16/CAG/0045, with public and patient involvement, and research ethics committee application: 16/SW/0088. It was registered with ClinicalTrials.gov: NCT02810860, and NUH research and innovation: 15GS002.

## Results

**Fig. 1:**
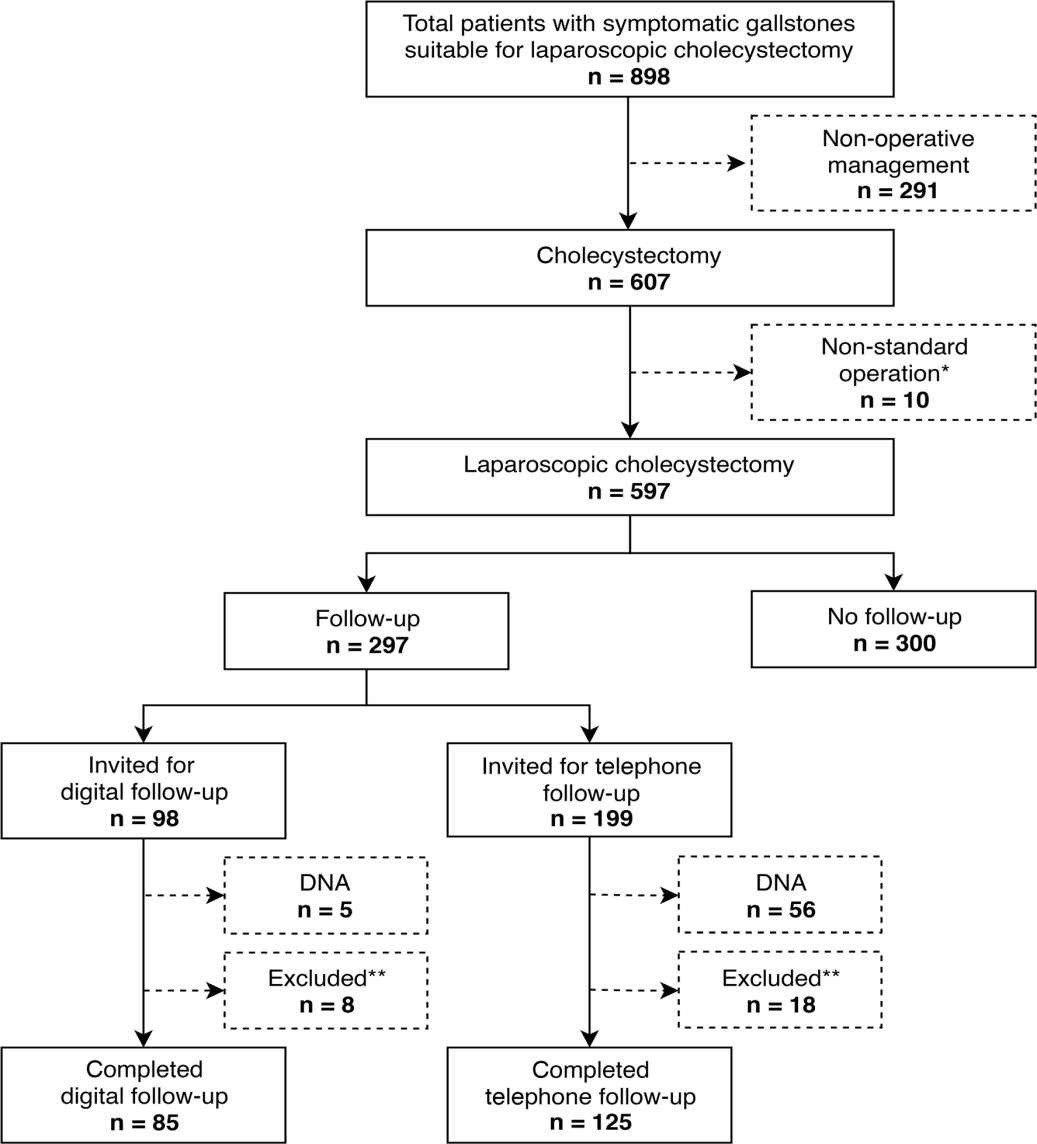
Participant recruitment (STROBE diagram). DNA: Did not attend (patient missed their appointment despite prompting). *Non-standard operation = open cholecystectomy, laparoscopic cholecystectomy combined with another operation such as liver resection or gastric bypass. **Excluded = follow-up performed at ≥ 31 days post-operatively

A total of 898 eligible patients were invited to participate in the study and use the digital platform across the three NUH sites. Some 607 (67.6%) patients went on to undergo cholecystectomy, with the remainder having non-operative management (Details and exclusions in Fig. 1).

### Patient recruitment

Half (49.7%) of all patients undergoing laparoscopic cholecystectomy were offered post-operative follow-up. One third (33%) were offered DFU and two-thirds (67%) were offered TFU.

Of the patients who opted for follow-up, significantly more patients missed their TFU than DFU appointments (30.9% [*n* = 56], vs. 5.6% [*n* = 5], *p* < 0.001).

### Patient demographics

Both follow-up and no follow-up groups were equally matched, as were DFU and TFU groups (Table 2).

**Table 2:** Demographics in participants offered follow-up versus those not offered follow-up

	Follow-up versus no follow-up			Digital versus telephone follow-up		
	Follow-up	No follow-up	*p* value	Digital follow-up	Telephone follow-up	*p* value
	*n* = 210	*n* = 300		*n* = 85	*n* = 125	
*Age (years)*						
Mean (SD)	46.8 (15.2)	48.8 (15.2)	0.140*	44.9 (14.6)	48.1 (15.6)	0.197*
IQR	35.0–59.0	35.9–59.7		32.5–55.3	35.4–1.0	
*Sex*						
Female	171 (81.4)	256 (85.3)	0.273^†^	66 (77.6)	105 (84.0)	0.280^†^
Male	39 (18.6)	44 (14.7)		19 (22.3)	20 (16.0)	
*CCI*						
0	162 (77.1)	229 (76.3)	0.968^‡^	71 (83.5)	91 (72.8)	0.186^‡^
1	25 (11.9)	35 (11.7)		14 (16.5)	32 (25.6)	
2	13 (6.2)	22 (7.3)		0	2 (1.6)	
≥ 3	10 (4.8)	14 (4.7)		3 (3.5)	7 (5.6)	
*BMI (kg/m*^2^)						
Median (IQR)	29.6 (25.9–34.5)	29.4 (25.9–34.7)	0.796*	29.0 (25.0–35.0)	30.1 (27.0–34.0)	0.170*
< 18.5	1 (6.8)	2 (0.7)	0.353^‡^	1 (1.2)	0	0.211^‡^
18.5–24.9	41 (19.5)	52 (17.3)		22 (25.9)	19 (15.2)	
25.0–29.9	65 (30.9)	103 (34.3)		24 (28.2)	41 (32.8)	
30.0–34.9	54 (25.7)	63 (21.0)		17 (20.0)	37 (29.6)	
35.0–39.9	30 (14.3)	55 (18.3)		13 (15.3)	17 (13.6)	
≥ 40.0	18 (8.6)	15 (5.0)		7 (8.2)	11 (8.8)	
Missing	1 (6.8)	10 (3.3)		1 (1.2)	0	
*LOS (days)*						
0	137 (65.2)	190 (63.3)	< 0.001^‡^	55 (64.7)	82 (65.6)	1.000^†^
1	68 (32.4)	70 (23.3)		28 (32.9)	40 (32.0)	
2	1 (0.5)	14 (4.7)		1 (1.2)	0	
≥ 3	4 (1.9)	26 (8.7)		1 (1.2)	3 (2.4)	

Values expressed as number (%), unless otherwise stated

*SD* standard deviation, *IQR* inter-quartile range, *CCI* Charlson Comorbidity Index, *BMI* Body Mass Index

*Mann–Whitney *U* test

^†^Fisher’s exact test

^‡^Chi-square test

### Follow-up versus no follow-up

#### 30-day complications

Significantly more complications were identified by patient follow-up than no follow-up (Table 3). In particular, the incidence of port-site wound infections and constipation were greater in the follow-up group.

**Table 3:** 30-Day complications identified by participant follow-up, compared with those seen in no follow-up group

	Follow-up versus no follow-up			Digital versus telephone follow-up		
	Follow-up *n* = 210	No follow-up *n* = 300	*p* value^†^	Digital follow-up *n* = 85	Telephone follow-up	*p* value^†^
					*n* = 125	
30-day complications	47^a^ (27.4)	27 (9.0)	< 0.001	15^b^ (17.6)	32^c^ (25.6)	0.183
Specific:	36 (17.1)	23 (7.7)	0.001	10 (11.8)	26 (20.8)	0.096
Intra-operative	0	1 (0.3)*	1	0	0	–
Common bile duct injury	0	1 (0.3)	1	0	0	–
Bile leak	0	1 (0.3)	1	0	0	–
Gallbladder bed collection	1 (0.5)	2 (0.7)	1	1 (1.2)	0	0.405
Acute pancreatitis	0	2 (0.7)	0.515	0	0	–
Port-site wound infection	27 (12.9)	7 (2.3)	< 0.001	5 (5.9)	22 (17.6)	0.012
Port-site wound dehiscence	0	4 (1.3)	0.147	0	0	–
Persistent post-operative pain	8 (3.8)	5 (1.7)	0.158	4 (4.7)	4 (3.2)	0.717
Generic:	11 (5.2)	4 (1.3)	0.015	5 (5.9)	6 (4.8)	0.76
Constipation	8 (3.8)	1 (0.3)	0.004	4 (4.7)	4 (3.2)	0.717
Diarrhoea	1 (0.5)	1 (0.3)	1	1 (1.2)	0	0.405
Other	2 (0.9)	2 (0.7)	1	0	2 (1.6)	0.516

Values expressed as number (%), unless otherwise stated

^†^Fisher’s exact test

*Small bowel injury

^a^Total of 63 complications, but 16 not identified at follow-up

^b^Total of 22 complications, but 7 not identified at follow-up

^c^Total of 41 complications, but 9 not identified at follow-up

#### 30-day readmissions and reoperations

Only 15 (7.1%) of all 63 complications in the follow-up group required attendance (including review in the surgical admissions unit), and 2 (0.9%) a return to theatre. Only 3 (1.4%) of the readmissions were identified at follow-up (Table 4).

**Table 4:** Readmission, reoperation and mortality in follow-up and no follow-up groups

	Follow-up versus no follow-up			Digital versus telephone follow-up		
	Follow-up *n* = 210	No Follow-up *n* = 300	*p* value	Digital follow-up *n* = 85	Telephone follow-up *n* = 125	*p* value
Total complications at 30-days Identified complications at 30-days	63 47	27 n/a	< 0.001^†^	22 (25.9) 15 (17.6)	41 (32.8) 32 (25.6)	0.183^†^
Total re-attendances at 30-days Identified readmissions at 30-days	15 (7.1) 3 (1.4)	24 (8.0) n/a	0.866^†^	6 (7.1) 3 (3.5)	9 (7.2) 0	1.000^†^
Length of stay						
(median days)	0	4	0.134*	0	0	0.908*
(IQR)	0–3.0	0.5–6.7		0–4.0	0–3.5	
Total reoperations at 30-days Identified reoperations at 30-days	2 (0.9) 0	5 (1.7) n/a	0.705^†^	1 (1.2) 0	1 (0.8) 0	1.000^†^
Mortality	0	0	–	0	0	–

Values expressed as number (%), unless otherwise stated

*Mann–Whitney *U* test

^†^Fisher’s exact test

### Digital follow-up versus telephone follow-up

#### 30-Day complications

Significantly more post-operative wound infections were identified by TFU than DFU (Table 3).

#### 30-Day readmissions and reoperations

Only 6 of all 22 complications (7.1% of the 85 DFU participants) in the DFU group required readmission, and 1 a return to theatre. Only 3 of the readmissions were identified at follow-up (Table 4). Conversely, 9 of all 41 complications (7.2% of the 125 TFU participants) in the TFU group required readmission, and 1 a return to theatre. None of these were identified at follow-up.

#### Complications not identified at follow-up

No complications were missed by either the DFU or TFU modalities. Complications not identified were those which occurred either before follow-up was offered or after follow-up was completed. Early complications (Participants 1, 2, and 8–15) occurred before follow-up, late complications (Participants 3–7, and 16) occurred after follow-up. Complications identified after follow-up were either not present at the time of follow-up (Participants 4, 6, and 7) or identified at the time of follow-up with patients given appropriate advice should the complication evolve with time (Participants 3, 5, and 16) (Table 5).

**Table 5:** 30-day post-operative complications not identified at follow-up

Participant	Complication	Complication identified on post-op day *X* =		Follow-up on post-op day *X* =	Outcome	Readmission LOS (days)
*Digital follow-up*						
1	Post-op bleed	1	before FU	14	Laparoscopy and washout	7
2	Persistent pain	3	before FU	31	Review and advice	0
3	Chest infection	13	after FU	12	Review and antibiotics	0
4	Stitch sinus	24	after FU	6	Review and stitch removal	0
5	Constipation	26	after FU	13	Review and laxatives	0
6	Wound infection	27	after FU	5	Review and antibiotics	0
7	Wound infection	29	after FU	8	Review and abscess drainage	0
*Telephone follow-up*						
8	Wound dehiscence	1	before FU	8	Review and wound closure	0
9	PONV	2	before FU	8	Review, fluids and antiemetics	2
10	Bile leak	5	before FU	12	Laparoscopy and washout	4
11	Collection	5	before FU	22	Review and advice	0
12	Pain	5	before FU	22	Review and antibiotics	3
13	Wound infection	7	before FU	23	Review and abscess drainage	4
14	Pain	10	before FU	23	Review and advice	0
15	Pain	13	before FU	16	Review and advice	0
16	Wound infection	13	after FU	12	Review and antibiotics	0

Post-op: post-operative, LOS: length of stay, FU: follow-up, PONV: post-operative nausea and vomiting

### Digital follow-up survey

Post-operative follow-up was completed significantly earlier with DFU then TFU (median 6 days [IQR 5–11 days] vs. 13.5 days [IQR 6.7–22 days], *p* = 0.001).

**Fig. 2:**
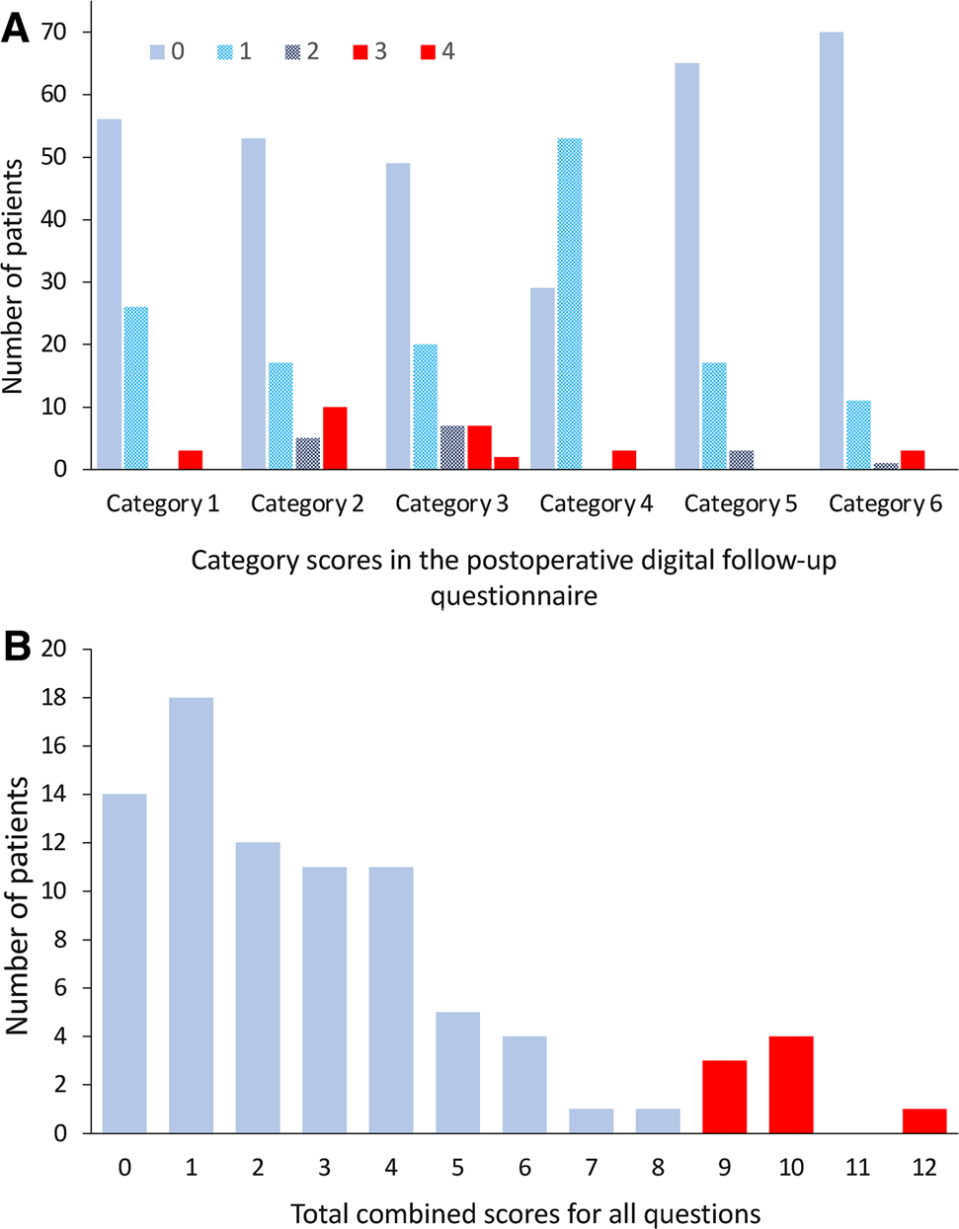
**a** Complications identified by digital follow-up survey: category scores. Cumulative scores for each category of the post-operative digital follow-up questionnaire. Scores ≥ 3 (shown in red) for each category triggered participants to have telephone follow-up. **b** Complications identified by digital follow-up survey: total scores. Cumulative scores for all 12 questions of the post-operative digital follow-up questionnaire. Scores ≥ 9 (shown in red) overall triggered participants to have telephone follow-up

A breakdown of DFU survey scores can be seen in Supplementary Table 1, with a summary of category scores in Fig. 2a and total scores in Fig. 2b.

All 15 participants who received a telephone call back following completion of their DFU survey expressed satisfaction in using the platform as an alternative to no follow-up or a physical follow-up. Comments included “removing the cost and need to find hospital parking”, “extra reassurance”, “having the option for downloadable recovery information”, and “being able to review information at any time”. Problems described by participants did not centre around the follow-up process but physical access to the online platform which provided the DFU. Specifically, problems were with regard to registration on the site and log-in which was not intuitive due to multiple security factors (email, password, two type-sensitive memorable security words).

## Discussion

This feasibility study has demonstrated that utilizing a DFU system is a viable alternative to TFU in participants undergoing elective laparoscopic cholecystectomy.

### Participant demographics

There were no statistically significant differences in participant variables between no follow-up and follow-up groups. Differences in total length of hospital stay after surgery could be explained due to differences in case complexity as the QMC site mainly has inpatient cholecystectomies performed by hepatobiliary surgeons. These participants with more technically difficult operations may experience a longer length of hospital stay, and in-hospital reviews; therefore, not warranting follow-up, or resulting in alternative follow-up arrangements not captured by this study. Unfortunately, operative difficulty was not collected as part of this study which would be a useful correlation [16], especially whilst analysing total length of hospital stay. The DFU and TFU groups were equally matched for participant and hospital specific variables.

### 30-Day complications

#### Follow-up versus no follow-up

As expected, follow-up groups had significantly more complications identified at 30-days post-operatively than no follow-up groups. This would coincide with the fact that in this observational study participants who received follow-up were not only actively tracked but also reviewed either by telephone consultation or in person. Thus, even the smallest of complications, such as surgical site infections and the development of constipation were logged and monitored. Conversely obtaining data on no follow-up participants was a little less transparent involving prospective audit whilst in hospital, and then monitoring of PAS and health care records for recorded planned and unplanned admissions and treatments. Resultantly more subtle complications may have been managed in the community and therefore missed for the purposes of this study.

#### Digital versus telephone follow-up

Participants who received TFU had greater recorded surgical site infections than those who underwent DFU. Although DFU participants were closely monitored throughout the study, not all DFU participants received a telephone interview and, therefore, the more subtle features of a wound infection may have been missed on our automated survey. However, many of the wound infections identified by TFU received only verbal advice on wound care as opposed to the necessity for hospital or general practitioner attendance or a course of antibiotics, and therefore the extra complications identified at TFU were potentially insignificant.

#### 30-Day readmission and reoperation

Overall numbers for readmission and reoperation at 30-days were small, and therefore although there were no significant differences between follow-up and no follow-up groups, the study was likely underpowered to comment. On review of national data however, 30-day readmission rates are quoted at 5.4–8.2%, and reoperation rates of 0.6–0.8% [2], exactly as seen in this study.

### Mortality

As expected, there were no deaths in any group. This is in keeping with mortality being a poor outcome measure for cholecystectomy research given its low incidence [2, 17, 18].

### Missed complications

No complications were missed nor were complications falsely identified in either DFU or TFU groups. This suggests that the automated DFU survey and associated scoring system is a safe alternative to conventional TFU.

The additional benefit of DFU is that the speed and ease of completion of follow-up surveys means that they could be offered at multiple post-operative time points to fully capture any possible early or late complications, such as those not captured in this study. Additionally, the digital process could be amended to facilitate a patient-triggered follow-up to aid earlier identification of those patients who may be developing a complication or need earlier clinician input. Such patient-triggered follow-up questionnaires could inform and give patients access to acute surgical triage units to ensure the timely admission and assessment of patients.

### Missed appointments

Participants who were offered DFU were significantly more likely to complete their follow-up than those offered TFU. This was even though participants in both groups had opted in for their chosen follow-up modality. Additionally, TFU participants had a pre-arranged date and time for their follow-up appointment which was not only confirmed on discharge but also posted to participants. Despite this, 30.9% versus 5.6% (*p* < 0.001) of participants missed their TFU compared with DFU appointments.

This may have been because DFU was the more convenient modality, as prior studies have commented on participants missing TFU appointments due to participants resting [4], incorrect telephone numbers [7], hospital admissions [7], or resuming their normal activities such as a return to work. Participants undergoing DFU also received an email informing them that their follow-up was due to be completed, and a further reminder if they had still not done so. This also meant that DFU was completed much earlier than TFU; a median of 6 days versus 13.5 days, respectively.

### Study strengths and limitations

Despite difficulties with participant recruitment and retention due to log-in difficulties required to complete the DFU survey, participants demonstrated a willingness to use DFU after laparoscopic cholecystectomy. Users described the availability of DFU and access to the digital platform as informative, reassuring, and convenient. Although it was not able to capture significant early complications it was able to capture feedback on more minor complications such as surgical site infections. In addition to providing useful feedback for surgeons, the timely management of these complications can also prevent more significant problems for patients. Patients with complications also reported improved care as completed DFU questionnaires triggered telephone appointments, discussion with specialists, and physical appointments without the trouble of arranging general practitioner appointments or walk-in centre waits.

Although this study provides practical, real-world information on the use of physician assisted DFU post-operatively, further research is necessary before its mainstream use, in particular a powered study to ensure that all groups are matched to ensure no missed complications. Due to third party data restrictions, we were unable to collect information on participant socio-economic status, which would have been useful to highlight any biases with DFU use.

### Future work

At a time where NHS resources are limited [19, 20], an automated follow-up process using DFU may reduce the need for resource intensive physical follow-up or TFU appointments.

Future work should compare matched groups of patients undergoing DFU, TFU, and no follow-up as a randomised controlled trial. Outcomes should include both quantitative and qualitative measures as this study but also consider patient satisfaction surveys, focus groups, and a cost analysis.

## Supplementary Information

Below is the link to the electronic supplementary material.

Supplementary file 1 (DOCX 15 kb)

## Declarations

### Data sharing

Data will be available upon reasonable request from Prita Daliya (prita.daliya@doctors.org.uk).
