# Supplementary material for: Digital Follow-Up After Elective Laparoscopic Cholecystectomy: A Feasibility Study
Source: World J Surg. 2022 Aug 11;46(11):2648–58. doi: 10.1007/s00268-022-06684-w (PMC9371370; doi:10.1007/s00268-022-06684-w)
Supplement: Supplementary file 1 — (DOCX 15 kb) [file 268_2022_6684_MOESM1_ESM.docx]

## Supplementary Table 1: Complications identified by digital follow-up survey.

| **Participant** | **Complication** | **Complication identified at follow-up on post-op day X =** | **Number of questions score >3** | **Overall survey score** | **Outcome** | **Readmission LOS** (days) |
| --- | --- | --- | --- | --- | --- | --- |
| 1 | GB bed collection | 7 | 2 | 9 | Admit & drain abscess | 3 |
| 2 | Wound infection | 8 | 1 | 7 | Telephone advice | n/a |
| 3 | Wound infection | 5 | 1 | 5 | Telephone advice | n/a |
| 4 | Wound infection | 11 | 2 | 10 | Admit & drain abscess | 0 |
| 5 | Wound infection | 15 | 3 | 12 | Admit & drain abscess | 0 |
| 6 | Wound infection | 6 | 1 | 6 | Telephone advice | n/a |
| 7 | Persistent pain | 5 | 1 | 9 | Telephone advice | n/a |
| 8 | Persistent pain | 7 | 1 | 5 | Telephone advice | n/a |
| 9 | Persistent pain | 6 | 1 | 3 | Telephone advice | n/a |
| 10 | Persistent pain | 21 | 2 | 10 | Telephone advice | n/a |
| 11 | Constipation | 6 | 3 | 10 | Telephone advice | n/a |
| 12 | Constipation | 5 | 1 | 4 | Telephone advice | n/a |
| 13 | Constipation | 5 | 2 | 10 | Telephone advice | n/a |
| 14 | Constipation | 5 | 1 | 6 | Telephone advice | n/a |
| 15 | Diarrhoea | 6 | 1 | 5 | Telephone advice | n/a |

GB: Gallbladder.
